# Notoginsenoside R1 Ameliorates Diabetic Retinopathy through PINK1-Dependent Activation of Mitophagy

**DOI:** 10.3390/cells8030213

**Published:** 2019-03-02

**Authors:** Ping Zhou, Weijie Xie, Xiangbao Meng, Yadong Zhai, Xi Dong, Xuelian Zhang, Guibo Sun, Xiaobo Sun

**Affiliations:** 1 Institute of Medicinal Plant Development, Peking Union Medical College and Chinese Academy of Medical Sciences, Beijing 100193, China; zhoup0520@163.com (P.Z.); xwjginseng@126.com (W.X.); 18210482526@163.com (X.M.); shengjupan@163.com (Y.Z.); dx5212004@126.com (X.D.); xlzhang2022@163.com (X.Z.); 2 Key Laboratory of New Drug Discovery Based on Classic Chinese Medicine Prescription, Chinese Academy of Medical Sciences, Beijing 100193, China

**Keywords:** diabetic retinopathy, mitophagy, PINK1, Notoginsenoside R1

## Abstract

Accumulating evidence has indicated that inflammation, oxidative stress, apoptosis, and autophagy in retinal Müller cells are involved in diabetic retinopathy (DR). Notoginsenoside R1 (NGR1), a novel saponin extracted from *Panax notoginseng*, posesses pharmacological properties, including treating diabetic encephalopathy and improving microcirculatory disorders. Nevertheless, its beneficial effects on DR and the potential mechanism remain to be elucidated. In this study, we found retinal vascular degeneration, reduced retinal thickness, and impaired retinal function in db/db mice were all dramatically attenuated by oral treatment with NGR1 (30 mg/kg) for 12 weeks. NGR1 pretreatment also significantly inhibited apoptosis, markedly suppressed the VEGF expression, markedly increased PEDF expression and markedly inhibited oxidative stress and inflammation in rat retinal Müller cells (rMC-1) subjected to high glucose (HG) and in the retinas of db/db mice. Furthermore, NGR1 pre-treatment upregulated the level of PINK1 and Parkin, increased the LC3-II/LC3-I ratio, and downregulated the level of p62/SQSTM1 in rMC-1 cells induced by HG and in the retinas of db/db mice. Moreover, NGR1 administration enhanced the co-localization of GFP-LC3 puncta and MitoTracker in rMC-1 cells. Importantly, knockdown of PINK1 abolished the protective effects of NGR1. In conclusion, these phenomena suggested that NGR1 prevented DR via PINK1-dependent enhancement of mitophagy.

## 1. Introduction

Diabetic retinopathy (DR), a severe complication of diabetes, continues to be the main reason for blindness in working-age individuals on a global scale [1]. The histological features of DR include the breakdown of the blood-retinal barrier, neovascularization, capillary non-perfusion, pericyte drop out, loss of endothelial cells, and relentless abnormal fibrovascular proliferation [2]. Although extensive research has been conducted, the pathophysiology of DR has not been fully elucidated. Numerous studies have suggested the fact that the pathogenesis of DR is involved in the functional disorder of Müller cells [3,4]. Müller cells maintain the balance between angiogenic factors, including vascular endothelial growth factor (VEGF), and antiangiogenic factors, including pigment epithelium-derived factor (PEDF) [5,6].

Although strict control of glycaemia, hypertension and hyperlipidaemia is still the main strategy in the primary treatment of DR, the recommended goals are difficult to achieve in many patients [7]. Current therapies for DR, including laser photocoagulation and anti-VEGF agents, significantly reduces the incidence of severe vision loss. However, existing therapies are not uniformly successful in halting visual decline, they are associated with troublesome side effects, and potentially serious complications may occur [8]. Therefore, novel alternative pharmacological therapies based on the pathophysiological mechanisms of DR are urgently needed in the clinic. Importantly, the major overlap that has been observed between traditional Chinese medicine (especially Panax notoginseng) and DR treatment may highlight additional therapeutic options for better managing DR [9,10].

A previous study reported the use of *P. notoginseng* from traditional Chinese medicine for the treatment of DR [11,12]. However, the mechanism of the effect of *P. notoginseng* remains unclear. NGR1 is a bioactive compound separated from *P. notoginseng*. Thus, the present study was designed to evaluate the beneficial effects and mechanism of NGR1 against DR in rat retinal Müller cells (rMC-1) exposed to high glucose (HG) and in the retinas of db/db mice.

Among the various biochemical pathways implicated in the physiologic abnormalities of Müller cells in DR, studies have focused on mitochondrial homeostasis and the signal transduction pathways needed to support mitochondria [13,14]. Mitochondria play a crucial role in the regulation of inflammation, oxidative stress, autophagy and apoptosis [15,16,17]. Damaged and dysfunctional mitochondria accumulate in the retina of diabetic patients and diabetic rodents [18,19]. The efficient and selective elimination of damaged and dysfunctional mitochondria is critical for maintaining mitochondrial homeostasis.

Mitophagy, a specialized form of autophagy, is considered the central mechanism in mitochondrial quality and quantity control [20,21]. To initiate mitophagy, PTEN-induced putative kinase protein 1 (PINK1) expression is elevated on the outer membrane of dysfunctional mitochondria where PINK1 simultaneously raises Parkin, the E3 ubiquitin ligase [22,23]. Parkin subsequently evokes ubiquitin chain formation on mitochondrial outer membrane proteins. Then, the autophagy receptors are recruited, such as p62/SQSTM1, and link to the LC3B II autophagophore to form autophagosomes [24,25]. In this respect, PINK1 might represent an attractive novel therapeutic target for intervention in DR.

Therefore, we sought to further determine whether PINK1-mediated mitophagy involves the mechanism associated with the protective effects of NGR1.

## 2. Reagents and Methods 

### 2.1. Materials

NGR1 (molecular weight = 933.15; purity > 98%) was purchased from Shanghai Winherb Medical S and T Development (Beijing, China). Dulbecco’s modified Eagle’s medium/F12 (DMEM/F12) and foetal bovine serum (FBS) were obtained from Gibco (Grand Island, NY, USA). 3-(4,5-Dimethylthiazol-2-yl)-2,5-diphenyltetrazolium bromide (MTT) and fluorescent dye JC-1 were acquired from Enzo Life Sciences (New York, NY, USA). ELISA kits for 4-hydroxynonenal (4-HNE), 8-hydroxy-2′-deoxyguanosine (8-OHdG) and protein carbonyl were acquired from Expandbio (Beijing, China). An Annexin V/propidium iodide (PI) kit, MitoTracker^®^ Red CM-H2XRos and a MitoSOX™ assay kit purchased from Invitrogen (Grand Island). A terminal deoxynucleotidyl transferase biotin-dUTP nick end labelling (TUNEL) detection kit was purchased from Roche Diagnostics GmbH, Mannheim, Germany. Cell protein extraction kits, PINK1 siRNA, and control siRNA were provided by Santa Cruz Biotechnology (Dallas, TX, USA). Bicinchoninic acid assay kits were purchased from Pierce Biotechnology (Waltham, MA, USA). The pCMV-G FP-LC3 expression vector was acquired from Cell Biolabs (San Diego, CA, USA).

### 2.2. Cell Culture and Drug

rMC-1 cells were acquired from American Type Culture Collection (ATCC). rMC-1 cells were cultured in DMEM/F12 supplemented with 5% FBS at 37 °C in an incubator. In all experiments, rMC-1 cells in the exponential phase were used. NGR1 stock solution (1 M) was stocked in DMSO. HG (60 mM) was prepared in distilled water followed by filtering. The indicated concentrations of NGR1 and HG were prepared immediately before use.

### 2.3. Analysis of Cell Viability

The cell viability of rMC-1 cells was evaluated by MTT chemosensitivity testing with a microplate reader (SpectraFluor, Tecan, Sunrise, Austria). Briefly, rMC-1 cells were cultured in 96-well plates at a density of 8 × 10^3^ cells/well followed by culturing for 24 h. The cells were pre-incubated with NGR1 and then induced by HG or co-incubated with NGR1 and HG. The control cells were incubated in DMEM/F12 that contained an equivalent concentration of DMSO (the highest concentration was less than 0.1%). The cells were treated with MTT operating fluid (1 mg/mL final concentration) at 37 °C for 4 h. Then, 100 μL of DMSO was used to replace the MTT reagent. Cell viability was reflected by absorbance, which was measured at 570 nm using a microplate reader (SpectraFluor) after 2 min of shaking. Cell viability was expressed as a percentage of the control value. Each experiment was performed in quintuplicate using three independent cultures.

### 2.4. Measurement of Mitochondrial Membrane Potential

JC-1 (Enzo Life Sciences International, New York, NY, USA) staining was conducted to evaluate the changes in mitochondrial membrane potential by flow cytometry analysis. rMC-1 cells (1 × 10^5^ cells/mL) were cultured in six-well plates and grown for 24 h. The cells were pre-treated with NGR1 (20 μM) for 24 h followed by exposure to HG (60 mM) for 48 h. Each group of cells was collected followed by incubation with JC-1 dye working fluid in the dark for 30 min at 37 °C. After rinsing twice, the stained cells were analysed using FACSCalibur (BD Biosciences, San Jose, CA, USA).

### 2.5. Detection of the Apoptosis Rate

We evaluated the proportions of viable and apoptotic cells in different treatment groups using the Annexin V/PI assay kits. rMC-1 cells (1 × 10^5^ cells/well) were planted in six-well plates. NGR1 (20 μM) was added to co-incubate with cells for 24 h followed by challenge with HG (60 mM) for 48 h. Then the cells were collected for conditioning with 1 × Annexin V working solution, supplemented with PI (1 μg/mL), avoiding light for 15 min. Thereafter, 300 mL of 1× binding buffer was added, and samples were mixed for analyze with a FACSCalibur flow cytometer (BD Biosciences). The results are expressed and analysed from three independent experiments.

### 2.6. Evaluation of DNA Fragmentation

Cell apoptosis was examined with a TUNEL staining kit in line with the recommended procedure (Roche Diagnostics GmbH, Mannheim, Germany). Briefly, after all processes, rMC-1 cells were washed with PBS, fixed in 4% buffered formaldehyde for 30 min and then incubated with a methanol solution with 0.3% H_2_O_2_. After rinsing with PBS, the cells were incubated with a permeabilizing solution containing 0.1% Triton X-100 for 10 min. Then, the TUNEL reaction mixture was prepared for incubation with the cells for 1 h at 37 °C in the dark. Thereafter, the rMC-1 cells were washed with PBS and counterstained with diamidino-2-phenylindole (DAPI). After washing with an equilibration buffer, photographs were acquired with a fluorescence microscope (Leica DM4000, Frankfurt, Germany).

### 2.7. Detection of Caspase-3 Activity

A fluorescence staining kit (BioVision, Milpitas, CA, USA) was used to detect the activation degree of caspase-3 in rMC-1 cells. Briefly, after all processes, 50 μL of precooled buffer was added to each group for 10 min; then, 50 μL of 2× reaction buffer (containing 10 mM dithiothreitol) and 5 μL of DEVD-7-amino-4-trifluoromethylcoumarin were prepared for incubation with the cells at 37 °C for 2 h. Fluorescence intensity was detected at 400 nm excitation wavelength and 505 nm emission wavelength. Three independent experiments were performed independently.

### 2.8. Transient Transfection

rMC-1 cells (1 × 10^5^ cells/well) were cultured in six-well plates followed by transient transfection for 48 h with PINK1 siRNA or corresponding control siRNA or pCMV-GFP-LC3 expression vector using the GeneJammer reagent (Agilent Stratagene, Palo Alto, CA, USA) in line with the indicated procedures. PINK1 silencing was determined by RT-PCR and Western blotting.

### 2.9. Animals

For all experiments, principles were followed to reduce the number of animals used and to minimize their suffering. The protocol was approved by the Laboratory Animal Ethics Committee of the Institute of Medicinal Plant Development, Peking Union Medical College, and conformed to the Guide for the Care and Use of Laboratory Animals (Permit Number: SYXK 2017-0020). Five-month-old db/db mice (BKS/DB−/−) and age-matched nondiabetic littermates (BKS/DB+/+, db/m) were purchased from the Animal Laboratory Center of Nanjing University. The temperature and humidity of the breeding environment were kept within the specified ranges. The mice were fed until they were 26 weeks old and then randomly assigned to the vehicle-treated db/m (n = 12), NGR1 (30 mg/kg/day)-treated db/m (n = 12), vehicle-treated db/db mice (n = 12), and NGR1 (30 mg/kg/day)-treated db/db group (n = 12). NGR1 was freshly prepared in saline and administered by gavage for 12 weeks. The vehicle-treated db/m and db/db mice were given the same amount of saline. The general health of the mice was carefully monitored, and no significant difference was found in body weight or food and water intake between the vehicle-treated and NGR1-treated groups. After being administered by gavage for 12 weeks, retinal function was assessed by detecting OCT and ERG; two days later, the mice were sacrificed for subsequent experiments.

### 2.10. Electroretinogram and Visual Evoked Potential

To detect retinal function of the mice, flash electroretinography (FERG), scotopic full-field electrophysiology, and flash visual evoked potentials (FVEPs) were recorded using the Visual Electrophysiology Instrument (OPTO-III, Optoprobe, Burnaby, BC, Canada, Canada). Briefly, the mice were anaesthetized with a mixture of ketamine (100 mg/kg body weight) and xylazine (10 mg/kg body weight) after overnight adaptation to the dark. The eyes of the mice were dilated with 1% tropicamide. For ERG recordings, the loop electrode was fixed on the corneal surface of the indicated eye. Needle electrodes were inserted under the skin of the groin and leg. To detect flash VEPs, a needle electrode was inserted under the skin between the two ears to replace the loop electrode. Black plaques were used to cover unstimulated eyes during the experiment. For FERG and FVEP recordings, stimulus production and data collection were carried out with the Visual Electrophysiology Instrument (OPTO-III, Optoprobe, Canada). Signals were amplified by 10,000 bandpass filtered (0.5–100 Hz) and digitized at 300 Hz with 12-bit resolution. Mice were tested at multiple flash intensities (3.0 cd.s.m^−2^, four times), and the stimulus interval was 15 s.

### 2.11. Optical Coherence Tomography

Retinal thicknesses of the mice were examined using optical coherence tomography (isOCT, 4D-ISOCT Microscope Imaging System, Optoprobe, Burnaby, BC, Canada). After anaesthetizing, the eyes of the mice were dilated with 1% tropicamide and coated with viscoelastic material to form a plano-concave lens. “TruTrack TM Active Eye Tracking” and “Automatic Real Time (ART)” technologies were used. The images were acquired with the optic nerve head centred on the corresponding box by altering the position and angle of the mice. Then, retinal thicknesses were analysed with software (version 2.0) from OptoProbe Research Ltd. 

### 2.12. Transmission Electron Microscopy Analysis

The ultrastructure of retinal Müller cells was analysed by transmission electron microscopy JEOL JEM1230 (JEOL Ltd., Tokyo, Japan). Briefly, the retinas were fixed in 2.5% glutaraldehyde overnight and then the samples were processed for 60 min with 0.1 M sodium cacodylate buffer supplemented with 1% osmium tetroxide. The retinas were then treated with 2% uranyl acetate for 30 min, stimulated in different gradient concentrations of ethanol, and then embedded in PolyBed 812 resin. Sections were obtained at 70 nm thickness followed by staining with Venable’s lead citrate to photograph using a transmission electron microscope (JEOL, Tokyo, Japan).

### 2.13. HE Staining

Cervical dislocation was used to euthanize the mice, the eyeballs were quickly removed and fixed in 4% paraformaldehyde, embedded in paraffin and sectioned (5 µm). The eye sections were stained with haematoxylin and eosin (H and E). Serial sections in close proximity (within 100 μm) to the optic nerve head were obtained to ensure the parallel comparison of different groups and digital images were captured under a light microscope (BX51, Olympus Corporation, Tokyo, Japan). The retinal thickness was determined in vertical sections by measuring the distance from the retinal pigment epithelium (RPE) layer to the top of the INL.

### 2.14. Determination of Mitochondrial ROS

The level of mitochondrial ROS in rMC-1 cells and the retinas were determined by the mitochondria-specific probe MitoSOX™ (Carlsbad, CA, USA) following the steps recommended. The cells were pre-incubated with NGR1 (20 μM) for 24 h and subjected to HG (60 mM) for 48 h. rMC-1 cells were trypsinized and collected. After anaesthetizing, mice were perfused transcardially followed by removing eyeballs and detached retinas. The retinal tissues were homogenized in PBS. Subsequently, the cells and the homogenates were pre-treated with MitoSOX™ (0.2 μM final concentration) at 37 °C avoiding light for 40 min. Images were obtained to evaluate fluorescence intensity using a fluorescence microplate reader. The excitation and emission wavelengths were 495 and 529 nm, respectively. 

### 2.15. ELISA

The levels of VEGF, PEDF, 4-HNE, protein carbonyl, 8-OHdG, and the inflammatory factors (MCP-1, TNF-α, IL-6, and ICAM-1) were measured by enzyme-linked immune sorbent assay (ELISA) kits following the manufacturer’s instructions. ELISA kits for 4-hydroxynonenal (4-HNE), 8-hydroxy-2′-deoxyguanosine (8-OHdG) and protein carbonyl were acquired from Expandbio (Beijing, China), and ELISA kits for VEGF, PEDF, and the inflammatory factors (MCP-1, TNF-α, IL-6 and ICAM-1) were acquired from Abcam (Cambridge, MA, USA). The equiponderant retinal tissues derived from each group were dissociated in RIPA supplemented with phosphatase and protease inhibitor. The homogenates were centrifuged at 12,000 rpm for 15 min at 4 °C, and the supernatants were collected for ELISA. The rMC-1 cells were harvested and dissociated in lysis buffer. The lysates were centrifuged at 20,000 rpm for 10 min to obtain the supernatant for ELISA.

### 2.16. Western Blotting

Western blotting was performed to evaluate the corresponding proteins. Briefly, proteins were obtained by cell or tissue protein extraction kits supplemented with protease inhibitor and phosphatase inhibitor (Roche, Penzberg, Upper Bavaria, Germany) and stored at 4 °C for 15 min and then centrifuged at 15,000 rpm for 20 min to acquire the supernatant containing protein. A BCA quantitative kit was used to measure the protein concentration in each sample. Thereafter, equivalent concentrations of protein samples from different groups were prepared for electrophoresis and then imprinted onto a membrane. Next, the membranes were blocked for more than 2 h in non-fat milk powder solution at approximately 25 °C followed by incubation in skimmed milk containing primary and secondary antibodies according to a certain ratio. Tris-buffered saline and Tween 20 (TBST) were used to wash the membranes for 15 min, which was repeated three times. Then, the bands were visualized using an enhanced chemiluminescence solution. Protein expression was observed using Molecular Imager Lab, and densitometric analysis was performed using Gel Pro software (version 6.3).

### 2.17. Statistical Analysis

All data are expressed as the mean ± standard deviation (SD). When the data were normally distributed, they were analysed by unpaired two-tailed Student’s *t* tests, multiple groups were analysed by one-way analysis of variance (ANOVA), and multiple groups with two variables were analysed by two-way ANOVA. Data with equal variances were analysed by post hoc Bonferroni’s test, and data with unequal variance were analysed by Dunnett’s T3 test. When the data were not normally distributed, nonparametric tests were used. A *p* value < 0.05 was considered significant.

## 3. Results

### 3.1. NGR1 Pre-Treatment Exerted a Positive Effect on HG-Induced Cell Death in rMC-1 Cells

**Figure 1:**
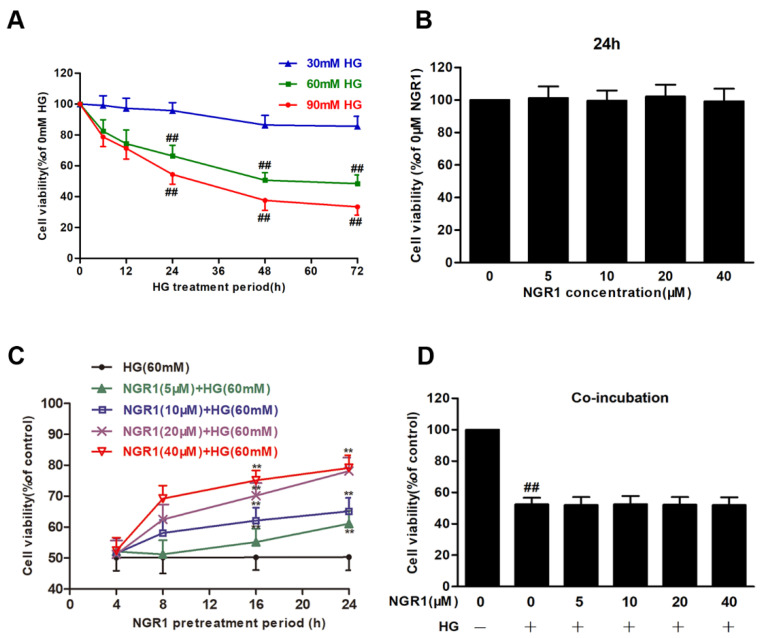
NGR1 preconditioning exerted a protective effect on HG-induced cell death in rMC-1 cells. Cell viability was tested by an MTT reduction assay. (**A**) HG increased cell death in rMC cells in concentration- and time-dependent manners. (**B**) NGR1 showed no effect on the cell viability of rMC cells. (**C**) NGR1 preincubation reversed HG-induced cell death in rMC cells in a dose- and time-dependent manners. (**D**) NGR1 had no protective effect when co-incubated with HG. The results were expressed as the means ± SD (n = 10). Two groups were compared by unpaired two-tailed Student’s *t* tests, and multiple groups were analysed by one-way analysis of variance (ANOVA); ## indicates a significant difference vs. control cells (*p* < 0.01). ** indicates significant difference vs. HG treatment (*p* < 0.01). (+), treatment with HG; (−), treatment without HG.

In our research, the effects of HG on rMC-1 cells were detected. rMC-1 cells treated with HG (30, 60 and 90 mM) for 12, 24, 48 and 72 h resulted in an obvious decline in cell viability in a time-dependent manner (Figure 1A). Treatment of rMC-1 cells with HG (60 mM) for 48 h reduced the cell viability to approximately 50% of the control cell viability (*p* < 0.01). Therefore, further experiments were performed using HG (60 mM) and a 48 h treatment period. In contrast, NGR1 had no effect on the cell viability of rMC-1 cells (Figure 1B; *p* > 0.05). However, NGR1 (5, 10, 20 and 40 μM) pre-treatment for 4, 8, 12 and 24 h significantly increased the cell viability of rMC-1 cells (Figure 1C; *p* < 0.01), followed by HG (60 mM) incubation. Unexpectedly, co-incubation of NGR1 (5, 10, 20 and 40 μM) with HG for 48 h led to almost no protection (Figure 1D; *p* > 0.05), which indicated that the protective function of NGR1 was conferred only when administered as a pre-treatment. In addition, to investigate whether 60 mM HG is toxic to cells due to osmotic pressure, mannitol was used as an osmotic control, and the effect of HG osmotic pressure on cells was separately investigated. No obvious toxicity was observed, and these data are provided in the Supplementary Materials (Figure S1).

### 3.2. NGR1 Inhibited HG-Induced Apoptosis in rMC-1 Cells

**Figure 2:**
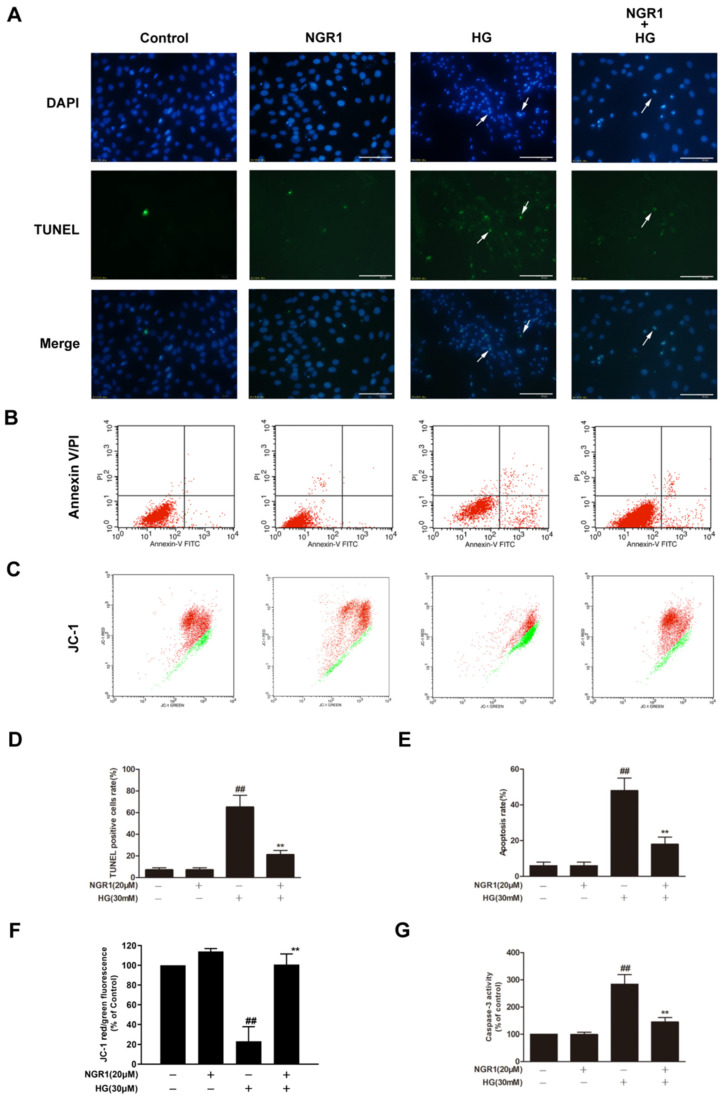
NGR1 preconditioning significantly inhibited HG-induced apoptosis in rMC-1 cells. NGR1 preconditioning attenuated HG-induced DNA fragmentation (**A**), Annexin V/PI double staining (**B**), and mitochondrial membrane depolarization (**C**) in rMC-1 cells. DNA fragmentation in rMC-1 cells was determined using TUNEL staining (bar = 100 μm). Apoptosis rate was quantified with Annexin V/PI double staining followed by flow cytometry analysis. Mitochondrial membrane depolarization was detected by JC-1 staining. The rate of TUNEL-positive cells (**D**), the quantification of Annexin V/PI double staining (**E**), and the percentage of JC-1 red to green fluorescence intensity (**F**) were quantitatively analysed, and caspase 3 activity (**G**) was detected by a fluorescence staining kit. The results are expressed as the means ± SD (*n* = 10). ## indicates a significant difference from control cells (*p* < 0.01). Two groups were analysed by unpaired two-tailed Student’s *t* tests, and multiple groups were analysed by one-way analysis of variance (ANOVA); ** indicates significant difference from HG treatment (*p* < 0.01). (+), treatment with HG or NGR1; (−), treatment without HG or NGR1.

DNA fragmentation, phosphatidylserine externalization, mitochondrial membrane potential loss and caspase-3 activation are characteristic features of rMC-1 cells undergoing HG-induced apoptosis. In the present study, HG-treated rMC-1 cells exhibited marked increases in the ratio of TUNEL-positive cells (Figure 2A,D; *p* < 0.01), the rate of Annexin V/PI double-labelled cells (Figure 2B,E; *p* < 0.01) and caspase-3 activity (Figure 2G; *p* < 0.01). Moreover, HG-treated rMC-1 cells exhibited a significant decrease in the percentage of JC-1 red to green fluorescence intensity (Figure 2C,F; *p* < 0.01). However, NGR1 administration notably reduced the ratio of TUNEL-positive cells and the rate of Annexin V/PI double-labelled cells, increased the percentage of JC-1 red to green fluorescence intensity and decreased caspase-3 activity in HG-treated rMC-1 cells (Figure 2; *p* < 0.01). The above phenomena indicate that NGR1 could prevent rMC-1 cell apoptosis induced by HG. Additionally, NGR1 administration alone showed no variation compared with control cells (*p* > 0.05).

### 3.3. NGR1 Significantly Attenuated DR in db/db Mice

**Figure 3:**
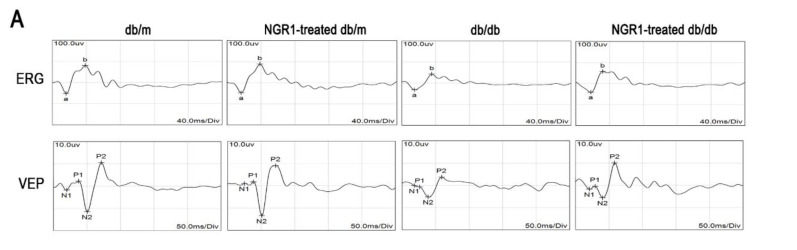

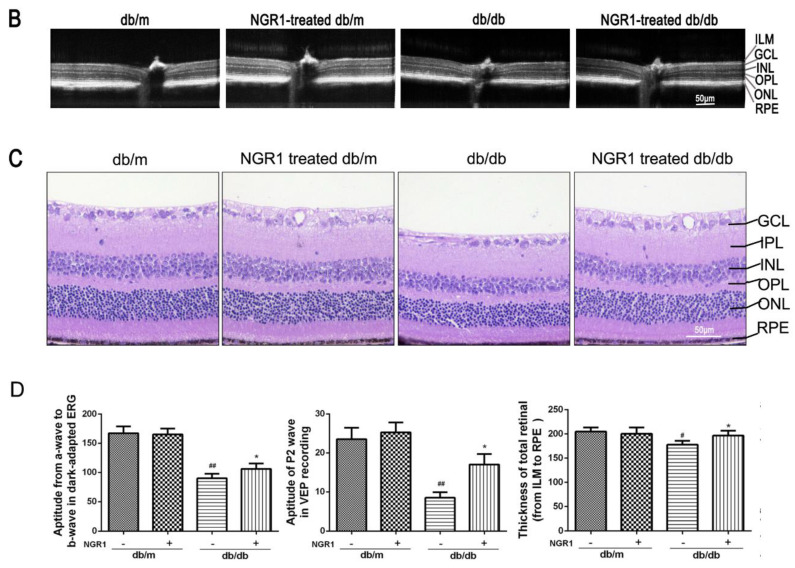
NGR1 pretreatment significantly attenuated DR in db/db mice. (**A**) Typical waveforms and quantitative analysis of ERG and VEPs amplitudes. (**B**) Retinal thickness was determined by OCT. (**C**) Retinal morphology was detected by HE. (**D**) Changes in retinal vasculature were monitored by FFA. (**E**) Corresponding statistics of ERG, VEPs, OCT and FFA. The number of mice in this experiment was 10 (n = 10). The results are expressed as the means ± SD. Two groups were analysed by unpaired two-tailed Student’s *t* tests, and multiple groups were analysed by one-way analysis of variance (ANOVA); # indicates significant difference from the control cells or db/m mice (*p* < 0.05); ## indicates a significant difference from control cells or db/m mice (*p* < 0.01). * indicates a significant difference from HG treatment or db/db mice (*p* < 0.05); ** indicates significant difference from HG treatment or db/db mice (*p* < 0.01). (+), treatment with NGR1; (−), treatment without NGR1.

Changes in visual functions, retinal thickness, and retinal vasculature were determined by ERG and OCT. As shown in Figure 3A, the ERG (from a wave to b wave) and VEP (P2) amplitudes were markedly decreased in db/db mice compared with db/m mice (*p* < 0.01). The amplitude of the a-wave or b-wave in db/db mice was significantly smaller than that in db/m mice, and was elevated in db/db mice by treatment with NGR1(Figure 3A, *p* < 0.05). OCT images showed that total retinal thickness, from the internal limiting membrane (ILM) to the RPE layer, was dramatically decreased in db/db mice (Figure 3B, *p* < 0.05). Treatment of db/db mice with NGR1 significantly affected the retinal thickness (Figure 3B, *p* < 0.05). Moreover, H&E staining showed that treatment of db/db mice with NGR1 for 3 months markedly increased the thickness of retinas, especially the ONL and the INL. The retinal morphology was similar between the db/m and NGR1-treated db/m groups (Figure 3C). The results of these experiments indicate that NRG1 improves retinal function and inhibits retinopathy in db/db mice.

### 3.4. NGR1 Reversed the Imbalance between VEGF and PEDF In Vivo and In Vitro

**Figure 4:**
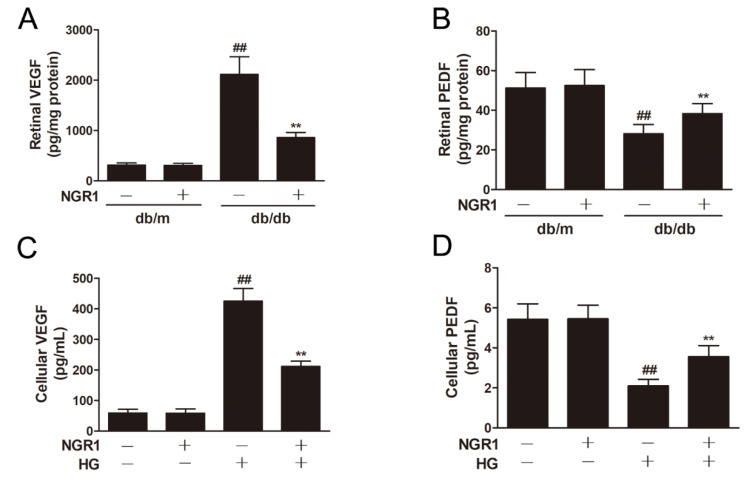
NGR1 pretreatment significantly attenuated the levels of VEGF and PEDF in vivo and in intro. The levels of VEGF (**A**) and PEDF (**B**) in retinas of db/db mice were determined by ELISA. The levels of VEGF (**C**) and PEDF (**D**) in HG-induced rMC cells were determined by ELISA. The results are expressed as the means ± SD (n = 10). Two groups were analysed by unpaired two-tailed Student’s *t* tests, and multiple groups were analysed by one-way analysis of variance (ANOVA); ## indicates a significant difference from control cells or db/m mice (*p* < 0.01). ** indicates a significant difference from the HG treatment or db/db mice (*p* < 0.01). (+), treatment with HG or NGR1; (−), treatment without HG or NGR1.

VEGF and PEDF play vital roles in the pathogenesis of DR. ELISA experiments demonstrated that HG treatment caused a notable upregulation in VEGF levels (Figure 4C; *p* < 0.01) and a remarkable decrease in PEDF levels (Figure 4D; *p* < 0.01) in rMC-1 cells. As expected, db/db mice showed a notable upregulation in VEGF levels (Figure 4A; *p* < 0.01) and a significant decrease in PEDF expression (Figure 4B; *p* < 0.01) in the retinas. However, NGR1 administration noticeably reduced the levels of VEGF and increased the levels of PEDF in HG-treated rMC-1 cells and in the retinas of db/db mice (Figure 4; *p* < 0.01).

### 3.5. NGR1 Inhibited Oxidative Stress In Vivo and In Vitro

**Figure 5:**
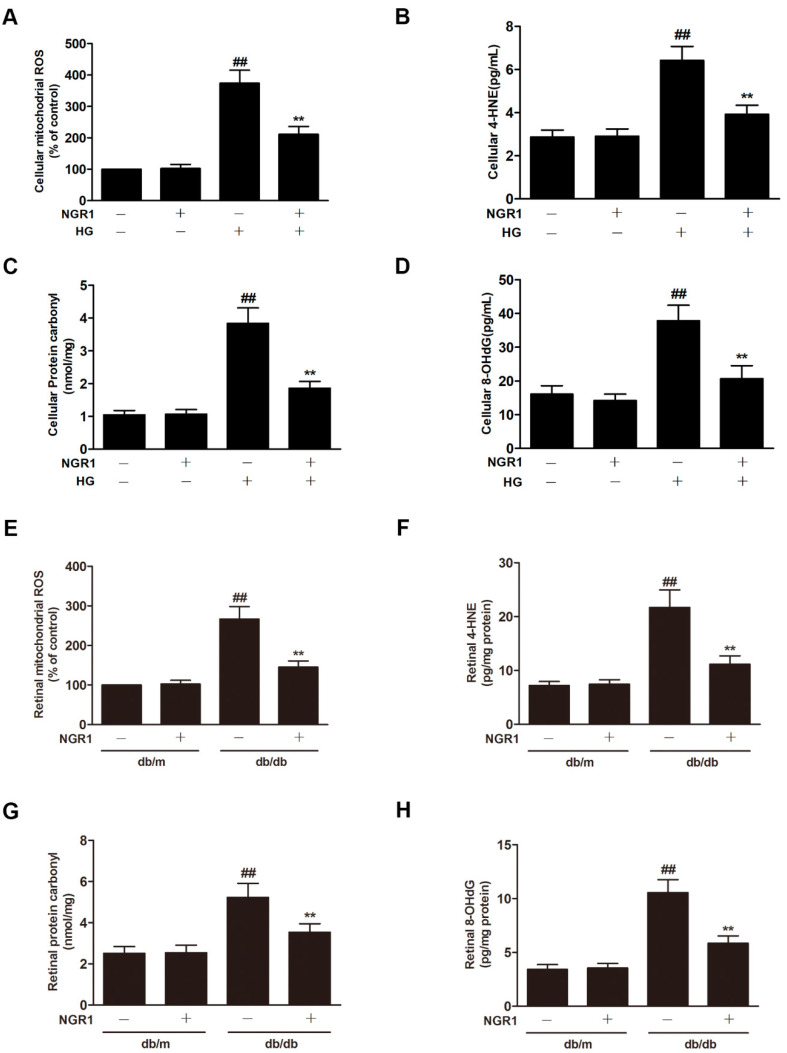
NGR1 pretreatment significantly suppressed oxidative stress in vivo and in intro. NGR1 preconditioning significantly suppressed HG-induced mitochondrial ROS production (**A**) and the production of 4-HNE (**B**), protein carbonyl (**C**), and 8-OHdG (**D**) in rMC cells. NGR1 pretreatment significantly decreased the level of mitochondrial ROS (**E**), 4-HNE (**F**), protein carbonyl (**G**), and 8-OHdG (**H**) in the retina of db/db mice. The level of mitochondrial ROS was determined by MitoSOX™. The production of 4-HNE, protein carbonyl, and 8-OHdG in rMC cells was detected by ELISA. The results are expressed as the means ± SD (n = 10). ## indicates a significant difference from the control cells or db/m mice (*p* < 0.01). ** indicates a significant difference from HG treatment or db/db mice (*p* < 0.01). (+), treatment with HG or NGR1; (−), treatment without HG or NGR1.

Oxidative stress plays an essential role in DR progression. Compared with control cells, HG caused a notable increase in the level of mitochondrial ROS (Figure 5A; *p* < 0.01) and in the levels of 4-HNE (Figure 5B; *p* < 0.01), protein carbonyl (Figure 5C; *p* < 0.01), and 8-OHdG (Figure 5D; *p* < 0.01) in rMC-1 cells. As expected, remarkable increases in the retinal levels of mitochondrial ROS, 4-HNE, protein carbonyl and 8-OHdG were observed in the retinas of db/db mice (Figure 5E–H; *p* < 0.01). However, NGR1 treatment substantially downregulated the levels of mitochondrial ROS, 4-HNE, protein carbonyl and 8-OHdG in HG-treated rMC cells and in the retinas of db/db mice (Figure 5; *p* < 0.01).

### 3.6. NGR1 Inhibited Inflammation In Vivo and In Vitro

**Figure 6:**
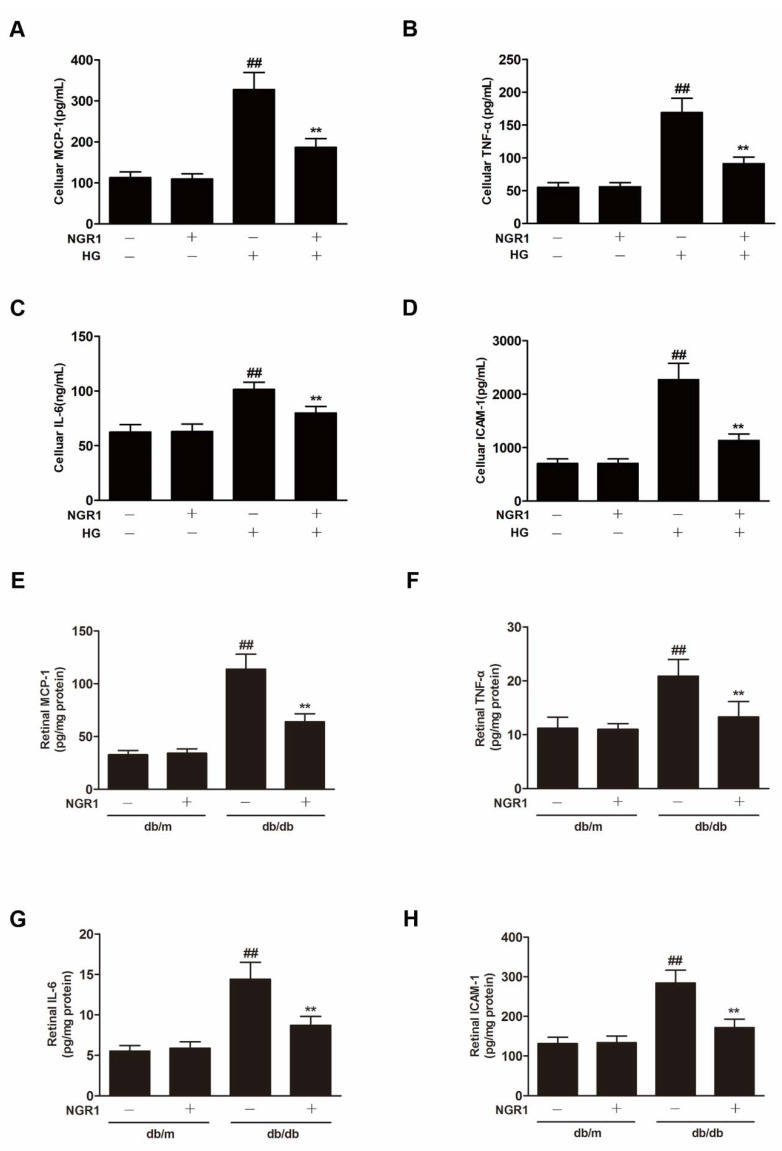
NGR1 pretreatment significantly inhibited inflammation in vivo and in intro. NGR1 preconditioning significantly inhibited HG-induced production of MCP-1 (**A**), TNF-α (**B**), IL-6 (**C**), and ICAM-1 (**D**) in rMC cells. NGR1 pretreatment significantly decreased the levels of MCP-1 (**E**), TNF-α (**F**), IL-6 (**G**), and ICAM-1 (**H**) in the retina of db/db mice. The levels of MCP-1, TNF-α, IL-6, and ICAM-1 were detected by ELISA. The results are expressed as the means ± SD (n = 10). ## indicates a significant difference from control cells or db/m mice (*p* < 0.01). ** indicates a significant difference from the HG treatment or db/db mice (*p* < 0.01). (+), treatment with HG or NGR1; (−), treatment without HG or NGR1.

In our study, HG exposure resulted in significantly increased inflammatory factor levels (MCP-1, TNF-α, IL-6 and ICAM-1) (Figure 6A–D; *p* < 0.01) in rMC-1 cells. The levels of inflammatory factors (MCP-1, TNF-α, IL-6 and ICAM-1) were significantly increased in the retina of db/db mice compared with db/m mice (Figure 6E–H; *p* < 0.01). NGR1 treatment inhibited the expression of inflammatory cytokines in HG-treated rMC cells and in the retina of db/db mice (Figure 6; *p* < 0.01).

### 3.7. NGR1 Enhanced *Mitophagy* In Vivo and In Vitro

**Figure 7:**
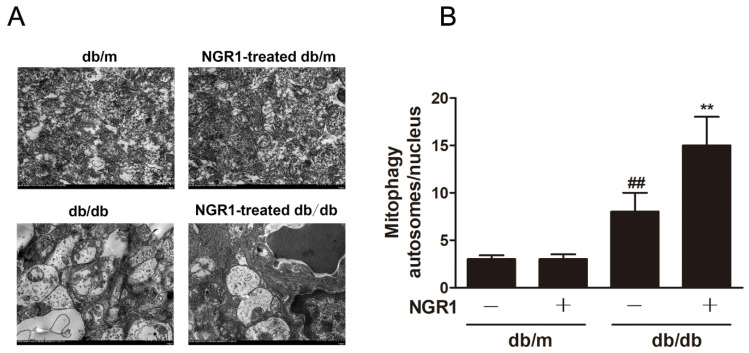
NGR1 pretreatment enhanced mitophagy in diabetic db/db mice with diabetic retinopathy. (**A**) The mitochondria in retinal Müller cells were analysed by transmission electron microscopy analysis. (**B**) Corresponding statistics of mitophagy autophagosomes. The results are expressed as the means ± SD (n = 5). They were analysed by unpaired two-tailed Student’s *t* tests, and multiple groups were analysed by one-way analysis of variance (ANOVA); ## indicates a significant difference from db/m mice (*p* < 0.01). ** indicates a significant difference from db/db mice (*p* < 0.01). (+), treatment with NGR1; (−), treatment without NGR1.

**Figure 8:**
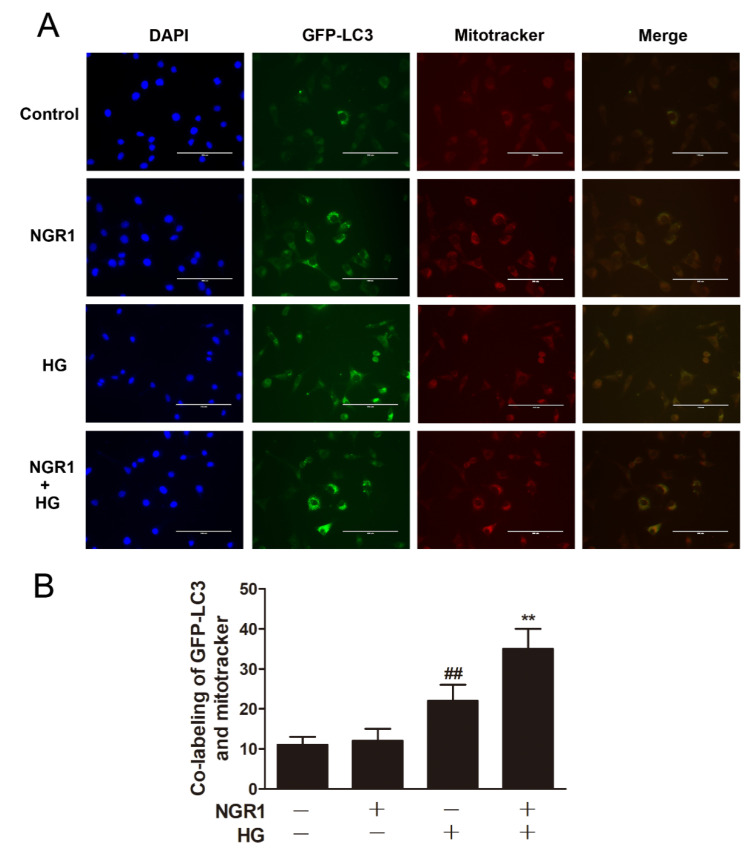
NGR1 pre-treatment enhanced mitophagy in HG-induced rMC1 cells. (**A**) rMC1 cells transiently transfected with the pCMV-GFP-LC3 expression vector were stained with MitoTracker^®^ Red CM-H2XRos (Bar = 100 μm). (**B**) Corresponding statistics of the GFP-LC3 expression level. The results are expressed as the means ± SD (n = 5). Two groups were analysed by unpaired two-tailed Student’s *t* tests, and multiple groups were analysed by one-way analysis of variance (ANOVA); ## indicates a significant difference from control cells (*p* < 0.01). ** indicates a significant difference from HG-treated cells (*p* < 0.01). (+), treatment with HG or NGR1; (−), treatment without HG or NGR1.

Strategies directed at enhancing mitophagy could have far-reaching beneficial effects. Therefore, we further investigated whether NGR1 affected mitophagy. As illustrated in Figure 7, compared with db/m mice, the number of mitophagy autophagosomes was elevated in the retinas of db/db mice (Figure 7, *p* < 0.01). However, more mitophagy autophagosomes were observed in NGR1-treated db/db mice (*p* < 0.01). Moreover, NGR1 enhanced mitophagy in rMC-1 cells, as revealed by the increased co-localization of GFP-LC3 puncta and MitoTracker^®^ Red CM-H2XRos in rMC-1 cells transiently transfected with the pCMV-GFP-LC3 expression vector (Figure 8, *p* < 0.01). 

**Figure 9:**
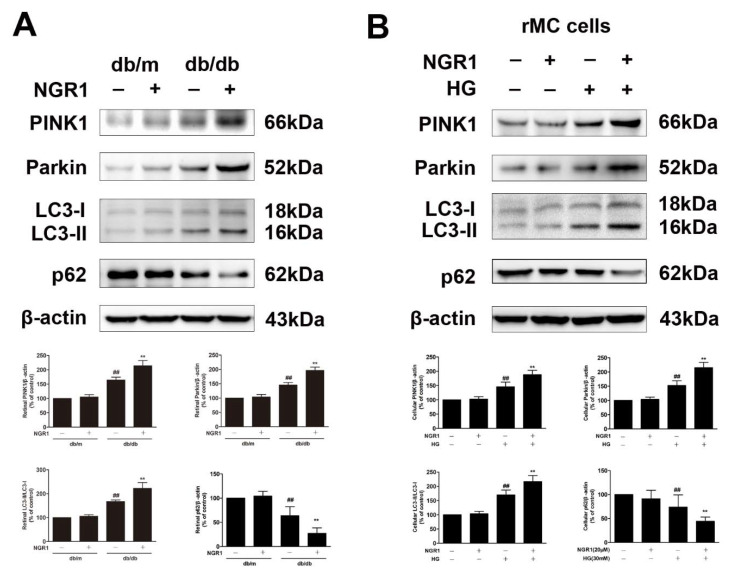
Effects of NGR1 pre-treatment on the expression level of mitophagy proteins in HG-induced rMC1 cells and in diabetic db/db mice with diabetic retinopathy. (**A**) PINK1, Parkin, LC3-II/LC3-I, and p62/SQSTM1 expression in the retina of db/db mice was determined by Western blotting. (**B**) The expression of PINK1, Parkin, LC3-II/LC3-I, and p62/SQSTM1 in HG-induced rMC1 cells was determined by western blotting. The results are expressed as the means ± SD (n = 10). Two groups were analysed by unpaired two-tailed Student’s *t* tests, and multiple groups were analysed by one-way analysis of variance (ANOVA); ## indicates significant difference from the control cells or db/m mice (*p* < 0.01). ** indicates a significant difference from HG treatment or db/db mice (*p* < 0.01). ## indicates a significant difference from control cells or db/m mice (*p* < 0.01). ** indicates a significant difference from HG treatment or db/db mice (*p* < 0.01). (+), treatment with HG or NGR1; (−), treatment without HG or NGR1.

The levels of PINK1 and Parkin and the ratio of LC3-II/LC3-I were dramatically increased in the retinas of db/db mice compared with the retinas of db/m mice (Figure 9, *p* < 0.01). However, NGR1 treatment even elevated the levels of PINK1 and Parkin and the ratio of LC3-II/LC3-I in the retinas of db/db mice (Figure 9, *p* < 0.01). To evaluate whether mitophagy functioned properly in db/db mice, the p62/SQSTM1 level was examined. The expression of p62/SQSTM1 was decreased in the retinas of db/db mice (Figure 9A, *p* < 0.01). Moreover, NGR1 pre-treatment even markedly downregulated the expression of p62/SQSTM1. In accordance with these results, compared with control cells, rMC-1 cells treated with HG exhibited noticeable increases in PINK1, Parkin, and LC3-II/LC3-I expression (Figure 9B; *p* < 0.01). NGR1 pre-treatment significantly enhanced the increased PINK1, Parkin, and the ratio of LC3-II/LC3-I in rMC-1 cells exposed to HG (Figure 9; *p* < 0.01). However, the decreased SQSTM1/p62 level induced by HG was further inhibited by NGR1 pre-treatment (Figure 9, *p* < 0.01).

### 3.8. NGR1 Enhanced Mitophagy by Activating PINK1

**Figure 10:**
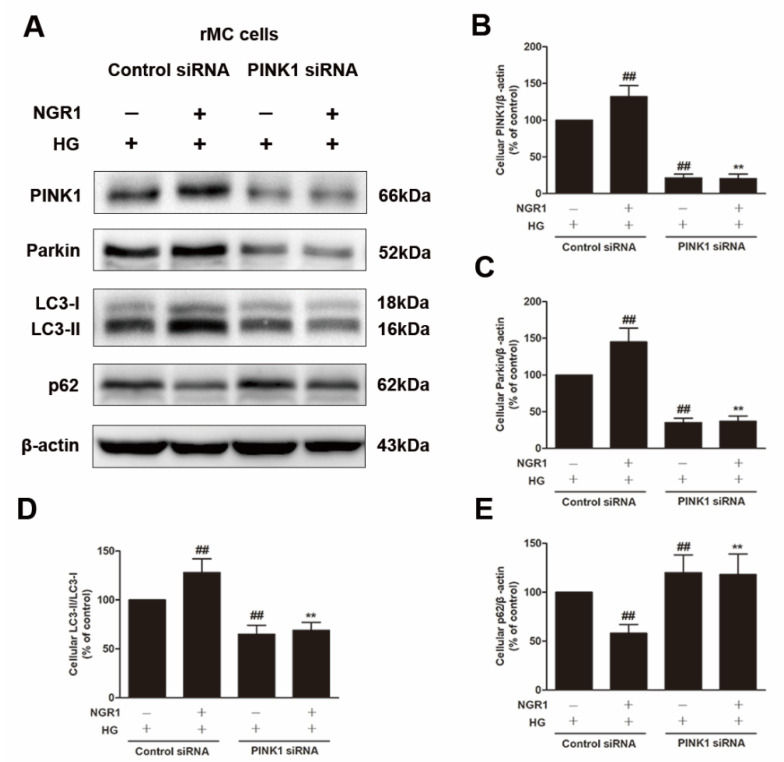
NGR1 enhanced mitophagy by activating PINK1. rMC cells were transiently transfected with PINK1 siRNA. (**A**) The expression of PINK1, Parkin, LC3-II/LC3-I, and p62/SQSTM1 in rMC cells was determined by Western blotting. (**B**) Quantitative densitometric analysis of PINK1 (**C**) and Parkin expression and (**D**) LC3-II/LC3-I ratio and (**E**) p62/SQSTM1 expression. The results are presented as a percentage of the control. The results are expressed as the means ± SD (n = 10). Two groups were analysed by unpaired two-tailed Student’s *t* tests, and multiple groups were analysed by one-way analysis of variance (ANOVA); ## indicates a significant difference from HG-treated cells transfected with control siRNA (*p* < 0.01). ** indicates a significant difference from cells in the NGR1+HG group. (+), treatment with HG or NGR1; (−), treatment without HG or NGR1.

**Figure 11:**
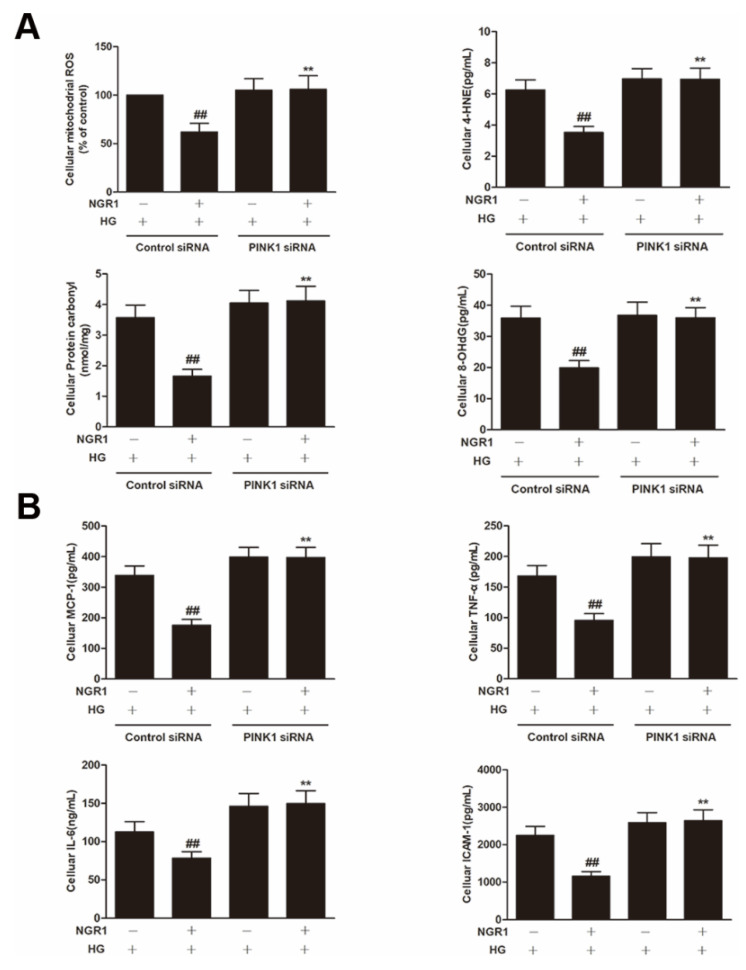
NGR1 suppressed oxidative stress inflammation by activating PINK1. rMC cells were transiently transfected with PINK1 siRNA. (**A**) The expression of cellular mitochondrial ROS, 4HNE, protein carbonyl and 8-OHdG. (**B**) The expression of the inflammatory factors MCP-1, TNF-α, IL-6 and ICAM-1. The results are expressed as the means ± SD (n = 10). Two groups were analysed by unpaired two-tailed Student’s *t* tests, and multiple groups were analysed by one-way analysis of variance (ANOVA); ## indicates a significant difference from HG-treated cells transfected with control siRNA (*p* < 0.01). ** indicates a significant difference from cells in the NGR1+HG group. (+), treatment with HG or NGR1; (−), treatment without HG or NGR1.

**Figure 12:**
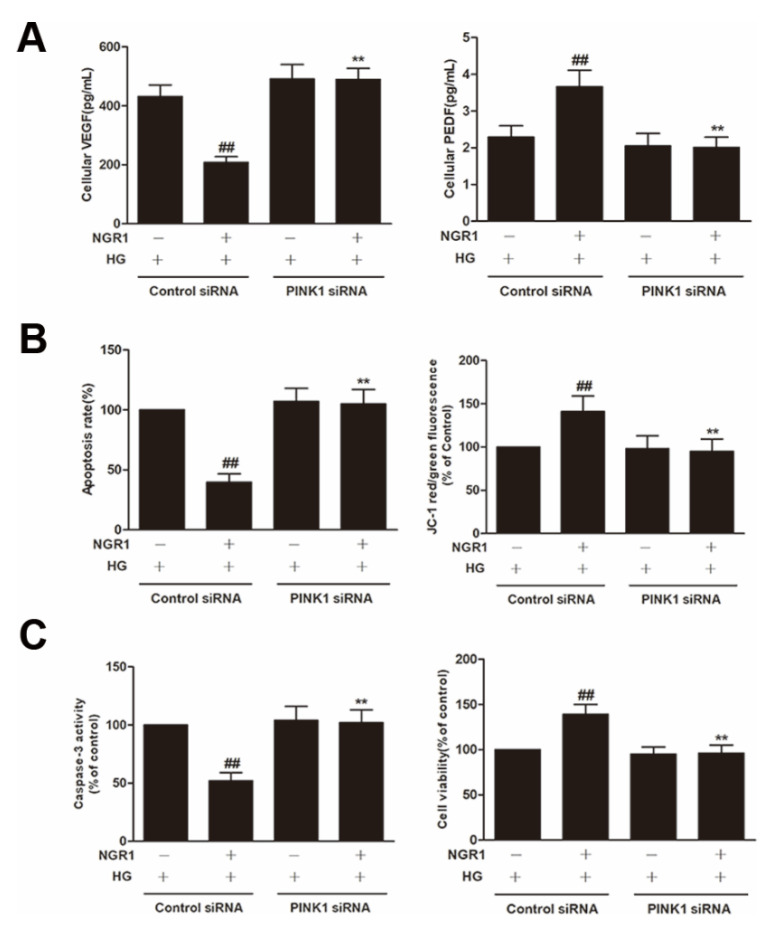
NGR1 attenuated apoptosis via activating PINK1. rMC cells were transiently transfected with PINK1 siRNA. (**A**) The expression of VEGF and PEDF; (**B**) the apoptosis rate and JC-1 ratio; (**C**) the caspase-3 activity and cell viability. The results are expressed as the means ± SD (n = 10). Two groups were analysed by unpaired two-tailed Student’s *t* tests, and multiple groups were analysed by one-way analysis of variance (ANOVA); ## indicates a significant difference from HG-treated cells transfected with control siRNA (*p* < 0.01). ** indicates a significant difference from cells in the NGR1+HG group. (+), treatment with HG or NGR1; (−), treatment without HG or NGR1.

To explore whether PINK1 was involved in NGR1-mediated mitophagy, rMC-1 cells were transiently transfected with PINK1 siRNA. As shown in Figure 10, PINK1 siRNA effectively reduced the expression of PINK1 and Parkin (*p* < 0.01) and abrogated the increased conversion of LC3-I to LC3-II induced by NGR1 (*p* < 0.01). Moreover, the suppression of oxidative stress (Figure 11A), the inhibition of inflammation (Figure 11B), the balance of VEGF and PEDF (Figure 12A), and the attenuation of apoptosis (Figure 12B,C) mediated by NGR1 were all abolished by PINK1 siRNA (*p* < 0.01).

## 4. Discussion

As a serious complication of diabetes, DR is one of the most common cause of visual impairment, and its prevalence has been increasing worldwide [26]. The current treatment approach for DR based on anti-oxidative, anti-inflammatory, and anti-angiogenesis drugs and laser photocoagulation is effective but also induces adverse effects in retinal tissues. Thus, a safe and effective mode of treatment is needed to control or delay DR. Based on previous evidence, treatment with natural anti-oxidant, anti-diabetic and anti-tumoral agents may be a promising therapeutic approach for the prevention of DR. NGR1 possesses a variety of pharmacological properties, including effects of cardiac protection and neuroprotection. This study was designed to evaluate the beneficial effects of NGR1 against DR in vivo and in vitro. We further sought to determine whether PINK1-mediated mitophagy is involved in the mechanism associated with the protective effects of NGR1.

In this study, NGR1 markedly increased the amplitudes of ERG and VEP, retinal thickness and markedly decreased the retinal vascular degeneration in db/db mice, which suggests that NGR1 significantly attenuated retinal dysfunction in db/db mice. In addition, the inner blood-retinal barrier (BRB) is a gliovascular unit in which macroglial cells surround capillary endothelial cells and regulate retinal capillaries by paracrine interactions. Previous studies suggest that Müller cells play a major role in the formation of barrier properties in retinal vessels [27]. Müller cells share the ability of astrocytes to induce the formation of barrier properties by vascular endothelial cells, and modulate vascular endothelial cells by express various cytokines, which, in turn, affects the retinal microvessels [28].

Studies have shown that during the pathogenesis of DR, long-term stimulation with HG can cause damage to Müller cells and induce Müller cell apoptosis, oxidative stress, inflammatory response, overexpression of VEGF [29,30]. In this study, Müller cells were simulated with concentrations of HG (30-60-90 mM) to evaluate the “in vitro” model. Treatment of rMC-1 cells with HG (60 mM) for 48 h reduced the cell viability to approximately 50% relative to the viability of control cells, resulting in oxidative stress, an inflammatory response and excessive production of VEGF. As these mechanisms of injury are consistent with the reported literature on diabetic pathology, we chose this concentration to mimic HG injury in vivo. However, some studies have found that diabetes can induce Müller cell activation and proliferation in db/db mice [31,32].The effects of HG on Müller cells at different stages of diabetes pathogenesis require further study. In addition, it has been reported that glial cells are activated in the hippocampus of diabetic mice and sustained HG stimulation leads to glial cell apoptosis [33], suggesting that in the retina, the state of glial cells may also change over time. 

Furthermore, Müller cells span the entire thickness of the retina, and contact and ensheath every type of neuronal cell body and process [34]. This morphological relationship is reflected by a multitude of functional interactions between neurons and Müller cells, including a “metabolic symbiosis” and the processing of visual information. Müller cells are also responsible for maintaining homeostasis of the retinal extracellular milieu [35]. Therefore, the thinning of the whole retina is most likely related to the morphology and function of Müller cells. Thus, in an in vitro model, we selected Müller cell damage induced by HG, and we found that NGR1 had an obvious protective effect.

In DR, retinas express low levels of PEDF and high levels of VEGF in experimental and clinical settings [36,37]. Therefore, VEGF and PEDF are now accepted as the key factors associated with DR and can constitute therapeutic targets. NGR1-treated db/db mice had a significantly elevated level of PEDF and a significantly decreased level of VEGF in retinal tissue. In parallel with these changes, NGR1 significantly increased the production of PEDF and decreased that of VEGF in HG-treated Müller cells. However, the molecular basis underlying these protective effects of NGR1 remains unknown. 

Several lines of evidence raise the possibility that mitochondria dysfunction in Müller cells plays pivotal roles in oxidative stress, inflammation, and apoptosis in the retinas of patients with DR [4,15,18]. Targeting mitochondrial homeostasis may confer advantages of inhibiting angiogenesis, oxidative stress, and inflammation, thereby effectively halting the development of DR. Thus mitochondria might be a potential therapeutic target for DR treatment. In this study, we observed that HG caused mitochondrial depolarization in Müller cells, resulting in increased mitochondrial ROS generation, oxidative stress and inflammatory cytokine production. Oxidative stress and inflammation were also observed in the retinas of db/db mice. The blockade of mitochondrial damage with NGR1 caused a significant blockage of oxidative stress and inflammation in HG-treated Müller cells and in the retinas of db/db mice.

PINK1 is a recently described regulator of mitophagy [38]. Mitophagy is a conserved multistep pathway that selectively degrades and recycles damaged mitochondria [39,40]. Therefore, stimulating PINK1-mediated mitophagy may represent a novel therapeutic strategy to prevent DR. NGR1 increased GFP-LC3 puncta and MitoTracker^®^ Red CM-H2XRos co-localization in HG-treated rMC-1 cells. To elucidate the involvement of PINK1 in NGR1-mediated mitophagy, PINK1 knockdown experiments were performed, and rMC-1 cells were transiently transfected with PINK1 siRNA. As shown in Figure 10, PINK1 siRNA abrogated the decreased p62/SQSTM1 induced by NGR1. However, the relationship between PINK and p62/SQSTM1 requires further detailed investigation. Our results show that PINK1 siRNA abolished the mitophagy mediated by NGR1. The suppression of oxidative stress, the balance of VEGF and PEDF, the inhibition of inflammation, and the attenuation of apoptosis mediated by NGR1 were all abolished by PINK1 siRNA. Based on the above results, the beneficial functions of NGR1 were PINK1-dependent. 

A recent study showed that the reduced PINK and Parkin expression noted in HK-2 cells subjected to HG exposure was partially restored by MitoQ, a mitochondria-targeted antioxidant. This effect was abolished by Nrf2 siRNA and augmented by Keap1 siRNA, which suggests that PINK and Parkin expression was regulated via Nrf2/Keap1 [41]. Similar results have been confirmed in various tumour cells [42]. In addition, studies have shown that AKT signalling can selectively regulate PINK1 mitophagy [43]. Previous studies by our team have revealed that R1 pre-treatment can activate the Nrf2 signalling pathway and affect the phosphorylation level of AKT in a diabetic mouse model [44]. Therefore, whether R1 plays an anti-DR role by activating the Nrf2 signalling pathway or PI3K/AKT and, thereby, regulating the PINK1/Parkin signalling pathway is worthy of further study.

Moreover, the administration method used in this study was oral administration. Compared to the intravitreal injection, oral administration has the advantage of being non-invasive and painless, and the main disadvantage of gavage administration is the uncertainty of the active metabolite of the drug. To date, no other studies have revealed the active metabolite of NGR1. Whether the active metabolites of NGR1 are the real contributors to the protective effect of NGR1 in vivo requires further detailed investigation.

In summary, the discovery of the PINK1 activator NGR1 not only provides a potential candidate drug for DR treatment but may also increase our understanding of the functions of PINK1 in the regulation of mitophagy in DR.

## Supplementary Materials

The following are available online at https://www.mdpi.com/2073-4409/8/3/213/s1, Figure S1: 60 mM HG preconditioning exerted no osmotic toxicity on rMC-1 cells.
