# Correction: Zhou, P., et al. Notoginsenoside R1 Ameliorates Diabetic Retinopathy through PINK1-Dependent Activation of Mitophagy. *Cells*, 2019, *8*, 213

**DOI:** 10.3390/cells9020450

**Published:** 2020-02-17

**Authors:** Ping Zhou, Weijie Xie, Xiangbao Meng, Yadong Zhai, Xi Dong, Xuelian Zhang, Guibo Sun, Xiaobo Sun

**Affiliations:** 1 Institute of Medicinal Plant Development, Peking Union Medical College and Chinese Academy of Medical Sciences, Beijing 100193, China; zhoup0520@163.com (P.Z.); xwjginseng@126.com (W.X.); 18210482526@163.com (X.M.); shengjupan@163.com (Y.Z.); dx5212004@126.com (X.D.); xlZhang2022@163.com (X.Z.); 2 Key Laboratory of new drug discovery based on Classic Chinese medicine prescription, Chinese Academy of Medical Sciences, Beijing 100193, China

The authors wish to make the following corrections to this paper [1]:

In the article “Notoginsenoside R1 Ameliorates Diabetic Retinopathy through PINK1-Dependent Activation of Mitophagy”, we found that not every batch of mice showed a similar FFA image in the subsequent experiment. Therefore, we decide to remove the contents (experiments and discussion) related to FFA measurement (original Figure 3D).

The corrected Figure 3 is shown as follows.

Simultaneously, relevant methodologies, conclusions and discussions are deleted.

In Section 2.11, on page 5, the following text is deleted: “2.11. Fundus Fluorescein Angiography. The retinal vascular changes of the mice were detected by fundus fluorescein angiography (FFA). After anaesthetising, the eyes of the mice were dilated with 1% tropicamide, and the eyeball was coated with viscoelastic material and covered with a coverslip to form a plano-concave lens. Fundus examinations were performed 2 min after intraperitoneal injection of 0.1 mL of 2.5% fluorescein sodium (Alcon, Houston, TX, USA) using a digital fundus camera (Retinal Imaging System, OptoProbe Research Ltd., Burnaby, BC, Canada) until the fifth minute. The fluorescence intensity of the photographs obtained from each group was analysed by using ImageJ software (version 1.8.0) for statistical analysis and comparison of the groups. The specific steps performed using the software were as follows: Open the image, Image→Type→8 bit→Adjust→Threshold, Analyse→Set measurements→Measure. ”

In Section 3.3, on page 9, the following text is deleted: “In addition, the FFA results showed that fluorescence leakage of microvessels and the formation of microangiomas in retinal were markedly increased in db/db mice compared with in db/m mice (Figure 3D; *p* < 0.01). However, treatment of db/db mice with NGR1 resulted in significant decreases in the leakage of retinal microvessels and the formation of microangiomas (Figure 3D, *p* < 0.01).”

In Section 4, on page 20, the following text is deleted: “Therefore, FFA was performed to observe microvessels changes under HG or R1 administration. A previous study showed no visible microvascular change by week 28 [29], our results suggested that fluorescence leakage of microvessels and the formation of microangiomas in the retinal were markedly increased in 38-week-old db/db mice compared with in db/m mice. As described by Bhatta, diabetic mice manifest significant retinal vascular degeneration at 15 months [30]. Therefore, different vascular states may be related to different time points of observation, the results of our study may further our understanding of the pathogenesis in the murine model of type 2 diabetes and provide a theoretical basis for clinical research.”

In addition, references [29] and [30] on page 23 are deleted.

The authors would like to apologize for any inconvenience this has caused the readers.

## Figures and Tables

**Figure 3:**
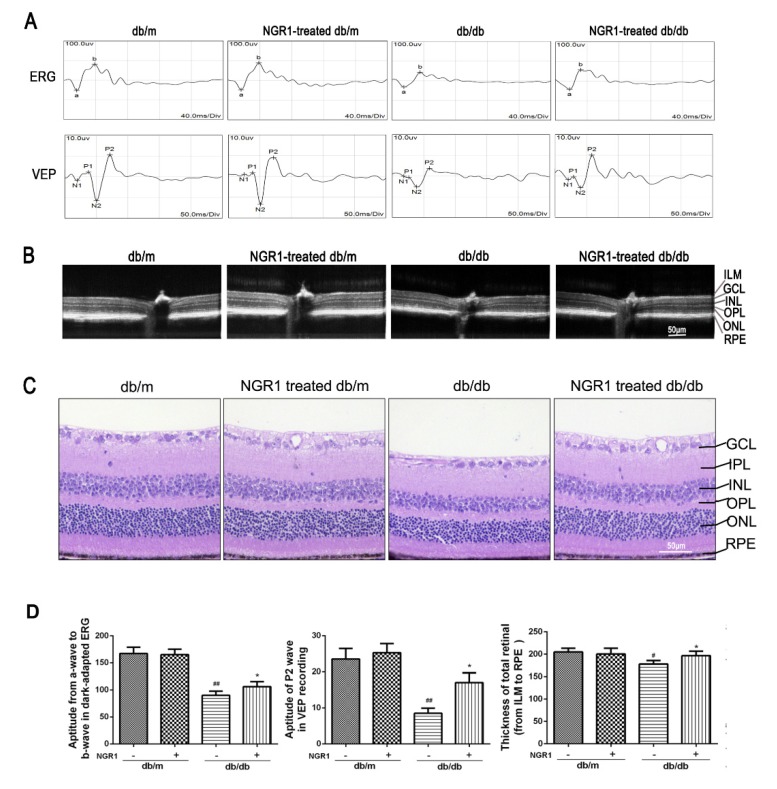
NGR1 pretreatment significantly attenuated DR in db/db mice. (**A**) Typical waveforms and quantitative analysis of ERG and VEPs amplitudes. (**B**) Retinal thickness was determined by OCT. (**C**) Retinal morphology was detected by HE. (**D**) Corresponding statistics of ERG, VEPs and OCT. The number of mice in this experiment was 10 (n = 10). The results are expressed as means ± SD. Two groups were analysed by unpaired two-tailed Student’s *t*-tests, and multiple groups were analysed by one-way analysis of variance (ANOVA); # indicates significant difference from the control cells or db/m mice (*p* < 0.05); ## indicates a significant difference from control cells or db/m mice (*p* < 0.01). * indicates a significant difference from HG treatment or db/db mice (*p* < 0.05); ** indicates significant difference from HG treatment or db/db mice (*p* < 0.01). (+), treatment with NGR1; (−), treatment without NGR1.
