# Correction: Zhou et al. Notoginsenoside R1 Ameliorates Diabetic Retinopathy through PINK1-Dependent Activation of Mitophagy. *Cells* 2019, *8*, 213

**DOI:** 10.3390/cells14080573

**Published:** 2025-04-11

**Authors:** Ping Zhou, Weijie Xie, Xiangbao Meng, Yadong Zhai, Xi Dong, Xuelian Zhang, Guibo Sun, Xiaobo Sun

**Affiliations:** 1 Institute of Medicinal Plant Development, Peking Union Medical College and Chinese Academy of Medical Sciences, Beijing 100193, China; zhoup0520@163.com (P.Z.); xwjginseng@126.com (W.X.); 18210482526@163.com (X.M.); shengjupan@163.com (Y.Z.); dx5212004@126.com (X.D.); xlzhang2022@163.com (X.Z.); 2 Key Laboratory of New Drug Discovery Based on Classic Chinese Medicine Prescription, Chinese Academy of Medical Sciences, Beijing 100193, China

In the original publication [1], there was a mistake in Figure 2 as published. In Figure 2C, the second and fourth panels were the same. The corrected Figure 2 appears below. The authors state that the scientific conclusions are unaffected. This correction was approved by the Academic Editor. The original publication has also been updated.

## Figures and Tables

**Figure 2:**
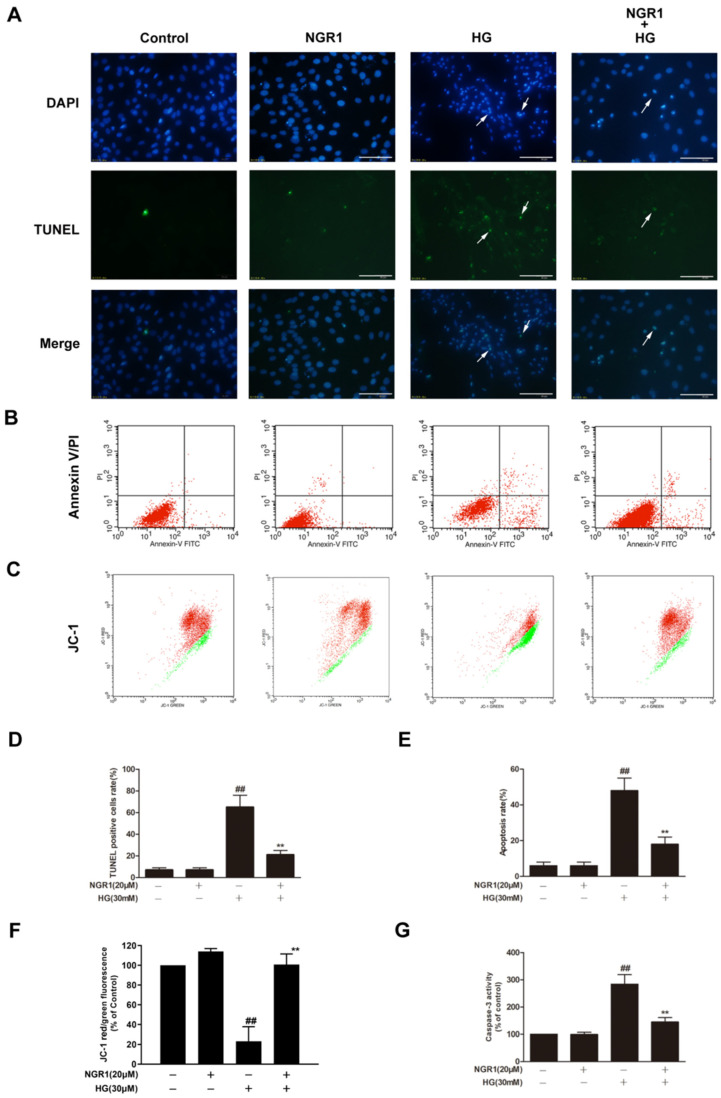
NGR1 preconditioning significantly inhibited HG-induced apoptosis in rMC-1 cells. NGR1 preconditioning attenuated HG-induced DNA fragmentation (**A**), Annexin V/PI double staining (**B**), and mitochondrial membrane depolarization (**C**) in rMC-1 cells. DNA fragmentation in rMC-1 cells was determined using TUNEL staining (bar = 100 μm). Apoptosis rate was quantified with Annexin V/PI double staining followed by flow cytometry analysis. Mitochondrial membrane depolarization was detected by JC-1 staining. The rate of TUNEL-positive cells (**D**), the quantification of Annexin V/PI double staining (**E**), and the percentage of JC-1 red to green fluorescence intensity (**F**) were quantitatively analysed, and caspase 3 activity (**G**) was detected by a fluorescence staining kit. The results are expressed as the means ± SD (*n* = 10). ## indicates a significant difference from control cells (*p* < 0.01). Two groups were analysed by unpaired two-tailed Student’s *t* tests, and multiple groups were analysed by one-way analysis of variance (ANOVA); ** indicates significant difference from HG treatment (*p* < 0.01). (+), treatment with HG or NGR1; (−), treatment without HG or NGR1.
